# Editorial: Occupational stress and joy of animal care professionals in zoos, sanctuaries, farms, shelters, and laboratory animal facilities

**DOI:** 10.3389/fvets.2023.1164483

**Published:** 2023-03-28

**Authors:** Sally Thompson Iritani, Sabrina Brando, Lynette Arnason Hart

**Affiliations:** ^1^Office of Research and Department of Environmental and Occupational Health Sciences, School of Public Health, University of Washington, Seattle, WA, United States; ^2^AnimalConcepts, Teulada, Alicante, Spain; ^3^Department of Psychology, University of Stirling, Stirling, United Kingdom; ^4^Department of Population Health and Reproduction, School of Veterinary Medicine, University of California, Davis, Davis, CA, United States

**Keywords:** compassion fatigue, empathic strain, burnout, mental health, caregiving, animal welfare

This compilation of articles is focused on the relationship between people and animals in different animal care working environments. The human–animal bond permeates and defines our relationships with animals. Many occupations involve regular daily interactions with animals that require not only providing for the basic needs of the animals but also supporting the animals in a scenario that necessitates understanding the animals' behavior and providing for their psychological wellbeing. One of the important recognized components is that we need to move from a culture of stoicism to a culture of compassion and feeling. This cultural shift will allow individuals to experience their emotions and provide a caring environment for both people and animals. We need to provide animal care professionals with resources and support to ensure that they can approach their jobs with resilience and provide optimal care for the animals.

## Impact on the animal care team

First and foremost, it is important to recognize that animal care is managed by a team of different professionals. This team consists of people with different attitudes toward and experiences of working with animals and people with different authority and decision-making regarding the care for the animals. Most animal care environments have primary caregivers who are responsible for the everyday needs of the animals, specialized teams that provide for the animals' health (clinical veterinary team) and wellbeing (psychological wellbeing and environmental surroundings), and high-level decision-makers such as directors of the zoos, sanctuaries, and farms. Each of these members will have a different experience of the joys and stressors related to their position; all team members, from frontline staff to the top leadership and CEO, should therefore be included and considered.

As such, Kogan et al. evaluated factors contributing to burnout and compassion fatigue, specifically for veterinary technicians on the team. Major factors that increased compassion satisfaction for these members were control over their schedules, recognition for their contributions, and opportunities for professional development. In addition, the authors identified that destigmatizing the “dirty work” would be helpful. This destigmatizing is important in other areas as identified in subsequent articles.

A particular stress of working with animals professionally is the requirement to deal with their deaths, often being in charge or participating in euthanasia, slaughter, or depopulation. The impact on the mental health of veterinary teams is a major focus of the American Veterinary Medical Association, which is building sources of support for those involved in the “Humane Endings” of animals in all contexts (1).

## Animal care professionals involved in care for shelter animals

Burnout and compassion fatigue for animal caregivers were identified early among animal caregivers that work in animal shelters (2). While the field is moving away from compassion fatigue to empathic strain, this editorial and Research Topic uses both interchangeably and is based on previous research.

Animal shelter workers have a critical public health role in assuring safety for both animals and humans, and they can experience great joy when placing an animal in the right environment and a new home for its future safety and overall wellbeing. These workers are also exposed to situations that can negatively impact their mental health at a higher frequency. This is attributed to the moral stress involved with decision-making relating to possible euthanasia and potential exposure to neglect, injury, or abuse of an animal. Hoy-Gerlach et al. make the case, in their eloquent overview of this situation, that there is an important role for social workers that are specifically trained and proficient at supporting and addressing the human-animal bond, compassion satisfaction, compassion fatigue, empathic strain, and burnout. The authors give several suggestions for forming a framework to support this endeavor that includes more specialized training and recognition from both social work societies and national animal care and control organizations to incorporate this into their strategic initiatives.

## Animal care professionals involved in care for research animals

Three of the articles in this Research Topic are focused specifically on the joys and associated sorrows of personnel that interact with research animals. This is another indicator of the amount of attention that is being devoted to recognizing and supporting animal caregivers in this profession. People that care for animals that are used in research are in a unique situation when it comes to the paradox of a caring profession. These jobs consist of providing daily care and oftentimes clinical care of animals that are used in scientific advancements to increase our understanding of both fundamental and applied biological sciences. There are associated challenges not seen in other animal care professions, in that there may be research protocols that involve creating an adverse effect on the animal and oftentimes at the end of an experiment the animals are euthanized for tissue collection and analysis.

LaFollette et al. have undertaken an extensive survey of a population of research animal care professionals and identified several areas for additional follow-up in evaluating the pervasiveness and contributing factors to compassion fatigue and compassion satisfaction. Compassion satisfaction was associated with higher social support, less animal stress/pain, and more human-animal interactions. In addition, a lower professional quality of life score was associated with the inability to provide more enrichment diversity at a greater frequency.

Murray et al. and Van Hooser et al. focused on intervention strategies for personnel that interact with research animals. Both articles make clear that the first step involves performing a needs assessment and determining what the organization needs to support its personnel. Murray et al. summarize two alternative approaches that can be tailored to an individual organization, depending on the size and complexity of the units involved. Van Hooser et al. summarize the approach used in a large academic institution. Importantly, the outcome is putting in place a compassion resiliency program that can support the needs of the organization and having a team approach that can ensure the sustainability of the program going forward.

## Summary

These articles touch on the joys and sorrows of animal caregivers in different environments. Clearly, many of these professions involve tremendous rewards and are also associated with both moral and compassion stress. Two factors identified will universally help in supporting animal care professionals. One is destigmatizing the work of our animal care professionals and providing sustainable support across our professions. The other is continuing to promote self-care, as explained in the articles, and seeking help and support if someone is feeling overwhelmed by their work. Finally, we would like to highlight the importance of attention to the individual, leadership, and organizational aspects of human wellbeing in these diverse settings, as all are equally essential.

## Author contributions

ST wrote the initial draft. LH and SB also contributed to the article and approved the submitted version.
